# Orthodontic Fixed Appliance and Periodontal Status: An Updated Systematic Review

**DOI:** 10.2174/1745017901814010614

**Published:** 2018-09-28

**Authors:** Silvia Cerroni, Guido Pasquantonio, Roberta Condò, Loredana Cerroni

**Affiliations:** Department of Clinical Science and Translational Medicine, University of Rome “Tor Vergata”, Rome, Italy

**Keywords:** Orthodontic therapy, Periodontal status, Fixed appliance, Systematic review, Orthodontics effects

## Abstract

**Background:**

Periodontal implications of orthodontic therapy are frequent, gingival and periodontal conditions need to be evaluated for every appointment. Several studies have analyzed the effects of fixed appliance on periodontal health.

**Objective:**

To evaluate whether there is updated scientific evidence on the relationship between fixed orthodontic therapy and periodontal health.

**Methods:**

A literature search was performed using the Pubmed and Cochrane databases and manual search; the search was carried out using the keywords “orthodontic” and “periodontal”. Articles published only in the English language from January 1997 to April 2017 were included. The inclusion criteria were: RCTs, cohort studies, cross-sectional studies and case-control studies only in English language; only studies on humans, with a minimum sample size of 20 patients and no restriction in terms of patient ages; orthodontic fixed appliances placed into the buccal tooth surface; standardization and training in oral hygiene; Periodontal Index (PI), Gingival Index (GI), Bleeding on Probing (BOP), Pocket Probing Depth (PPD), at least at baseline (before appliance was placed) and after follow up (with a minimum period of 3 months). The exclusion criteria were as follows: absence of baseline data before fixed appliances was placed; patients with systemic diseases, periodontal disease or craniofacial anomalies; removable appliances or orthodontic appliance on the lingual dental surface; and no standardization or training in oral hygiene.

Studies were selected by abstract and title; then, inclusion and exclusion criteria were applied. The studies that satisfied the inclusion criteria were evaluated and classified as having low, moderate or high methodology quality.

**Results:**

Fifty-five records were reviewed on the basis of title and abstract. After full-text reading, 47 full texts were excluded, and 3 articles were classified as having low methodological quality and 5 as having moderate methodological quality.

**Conclusions:**

The present systematic analysis suggests that there is moderate scientific evidence that a fixed appliance influences periodontal status; no article reported a high score.

## 1 INTRODUCTION

Orthodontic treatment is universally recognized as essential in the treatment of malocclusions. Proper therapy improves the occlusal and jaw relationship, masticatory function and facial aesthetics.

The effect of orthodontic therapy on periodontal status has been analyzed; small detrimental effects to the periodontium were highlighted [1].

Plaque retention during the use of a fixed orthodontic appliance has been determined to be an important etiological factor in the development of demineralization and chronic hyperplastic gingivitis [2]. Bogren *et al.*, suggested that the accumulation of microbial plaque on teeth is a direct cause of gingivitis and periodontitis [3].

The main components of fixed appliances (brackets, band, ligature and orthodontic wire) are able to reduce the physiological mechanism of self-cleaning by the tongue or cheeks, to increase retention of bacterial plaque and to change the bacterial population from a qualitative and quantitative point of view. In an *in vivo* study, an elastomeric ligature showed low susceptibility to plaque adhesion compared with the stainless steel of a metallic ligature [4].

The influence of fixed appliances on the quantity and quality of oral microbiota might be a transitional effect that depends on oral hygiene control [5].

However, dental alignment facilitates bacterial plaque removal and reduced occlusal trauma. Glans *et al.*, examined the relationship between crowding and gingival health during fixed orthodontic treatments; the study showed that orthodontic realignment enabled the patients to better perform techniques in oral hygiene [6].

Several studies have analyzed the correlation between a fixed appliance and the development of periodontal disease, but currently, only a few systematic reviews describe the results. The aim of the present study was to perform an updated systematic review to estimate the association between a fixed orthodontic treatment and periodontal status.

## 2 MATERIALS AND METHODS

The Pubmed and Cochrane databases were searched from January 1997 to April 2017 to find published studies on the effect of fixed appliances on periodontal status. The keywords used in the preliminary search were as follows “orthodontic” AND “periodontal”. The selection included all studies conducted on humans in the English language that investigated the effect of orthodontic fixed therapy on periodontal health. The review process, including search and selection (identification, screening, eligibility of included studies), was performed according to the PRISMA criteria [7].

In the selection process, all articles were selected by abstract and title; abstracts were initially read by two independent researchers to identify potentially eligible full-text papers. Then, inclusion and exclusion criteria were applied, and the studies were evaluated and classified. Duplicate papers were removed, and discrepancies between the two investigators were solved by discussion.

The following inclusion criteria were applied in our review:

### 2.1 Study Design

Randomized Controlled Trials (RCTs), cohort studies, cross-sectional studies and case control studies only in the English language.

### 2.2 Population

Only studies on humans, with a minimum sample size of 20 patients and no restriction in terms of patient ages.

### 2.3 Intervention

Orthodontic fixed appliances placed into the buccal tooth surface, standardization and training in oral hygiene (oral hygiene instructions).

### 2.4 Types of Outcome

Periodontal Index (PI), Gingival Index (GI), Bleeding on Probing (BOP), Pocket Probing Depth (PPD), at least at baseline (before appliance were placed) and after follow up (with a minimum period of 3 months).

**Fig. (1):**
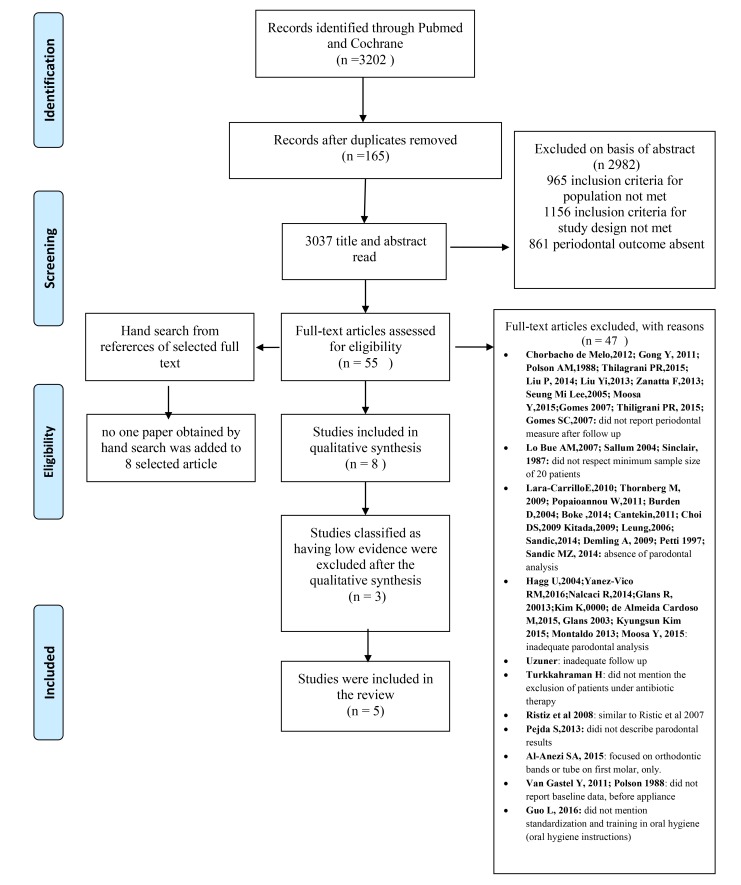
PRISMA Flow-chart of the screening of publications.

The exclusion criteria were as follows: absence of baseline data before fixed appliances was placed; patients with systemic diseases, periodontal disease or craniofacial anomalies; removable appliances or orthodontic appliance on the lingual dental surface; and no standardization or training in oral hygiene. Then, full-text articles were read, and the studies that satisfied both inclusion and exclusion criteria were carefully examined and qualified according to their methodological aspects, as described in (Fig. **1**) and Table **1**.

A modified checklist for assessing the quality was employed for this review [5].

For the manual search, we have selected seven journals (Journal of the American Dental Association, American Journal of Orthodontics and Dentofacial Orthopedics, Journal of orthodontic science, Angle Orthodontist, Orthodontics & Craniofacial Research, European Journal of Orthodontics, Journal of Periodontology), and search studies that investigate the association between periodontal health and fixed orthodontic therapy, in addition, the references of the selected articles were evaluated to find additional publications by manual searches.

Each article was assigned a final score; then, the score of each item was summed and classified according to the following classification according to Freitas *et al.*, [5]:

- Low (score from 0 to 5.9)
- Moderate (score from 6 to 8.9)
- High (scores 9 and 10).

Articles that were assigned a low final score were removed.

## 3 RESULTS

The aim of the present study was to evaluate the periodontal status in terms of the periodontal parameters after bracket and band placement. The electronic search identified 3,202 citations. Citations that were not connected with the topic were rejected. Title and abstract were selected according to the inclusion and exclusion criteria; the articles that presented at least one inclusion criteria and no exclusion criteria in the abstract were preserved. Studies evaluating both periodontal and microbiological analyses were included in this stage. Duplicates were considered only once. After evaluation, 55 records were screened on the basis of title and abstract.

Fifty-five full texts were read and analyzed, 47 that did not have appropriate full texts were excluded at this stage.

A manual search was performed with the references of the 55 full texts to find supplementary articles. Consequently, 17 new titles were considered; of these, 12 were excluded after reading the abstract and 5 were excluded after reading the full text. Thus, no paper obtained by the manual search was added to the 8 selected articles.

Therefore, 8 selected papers were examined and then classified according to the quality assessment that was previously described (Table **2**). Three articles were assigned a low final score and were excluded from this systematic review. Five articles were ranked as having moderate scientific evidence and were included in the review. Detailed quality information of studies included in the review is described in Table **3**. In Table **4**, the data of periodontal parameters extrapolated from selected 5 articles are reported.

## 4 DISCUSSION

Periodontal complications are reported to be one of the most common side effects linked to orthodontics [8]. The main complications associated with orthodontic fixed appliances are gingivitis, periodontitis, gingival recession or hypertrophy and alveolar bone loss [9, 10]. The presence of plaque is considered to be one of the main factors in the development of gingivitis [11, 12]; plaque retentive properties of the orthodontic appliance most likely cause an increase in plaque accumulation and gingival inflammation [13]. A rough surface and a gap at the composite enamel surface may cause plaque accumulation [14]; An excessive quantitative amount of composite around the bracket makes oral hygiene practices more difficult. The increased pathogenicity of plaque during orthodontic therapy has been described by several authors [15, 16, 1].

The aim of the present review was to evaluate the periodontal status in terms of the periodontal index after bracket and band placement. Microbiological results were not considered. Only studies on fixed appliances were selected, and removable appliances or lingual techniques were excluded. The main limitation of the review is the absence of a control group in all of the selected studies; a control group is important to account for periodontal changes in untreated subjects. Van Gastel reported that control sites (teeth that were not bonded/banded) did not show any significant change; this finding might be an indication that changes after bracket placement are local events [17].

Studies with a low methodological quality score were excluded from the review; none of these papers reported the study design, and the follow-up period did not last for more than three months [18-20]. The sample size was not large in any of the studies.

The articles included in this review were ranked as having moderate methodological quality. Three of them were conducted by the same authors [17, 21, 22], and two of them presented the same sample group and objectives but different follow-ups [21, 22], the most recent study reported 2 years of follow-up. Both papers were included in this review to compare the different follow up results. Only one article reported all four periodontal parameters: GI, PI, PPD and BOP [13]. All articles except one described the study design and the ethical aspects of the research. All studies described the sample standard participants and sample characterization; no study reported a control group or sample size calculation.

Ristic *et al.*, investigated the clinical and microbiological effects on periodontal tissue in adolescents. The periodontal index (PI, GI, GBI and PPD) and microbiological parameters were determined before appliance placement and 1, 3 and 6 months after the beginning of treatment. The results described an increase in all clinical and microbiological parameters after appliance placement; maximum values were obtained after 3 months. Subsequently, the periodontal scores decreased 6 months after fixed appliance placement. The authors concluded that the fixed appliance may increase all periodontal values; however, fixed appliances do not have a destructive effect because of transient conditions [13].

In 2008, Jan van Gastel *et al.*, evaluated the clinical and microbiological changes after bracket and band placement. In this study, 24 subjects were treated with headgear and received bands 18 weeks prior to receiving bonding brackets 10 subjects were treated with brackets only. In the headgear group, a comparison between the bonded and banded sites was possible. Periodontal index (PPD and BOP) and microbiologic analysis were measured at baseline (band placement), at weeks 18 (bracket placement), 20, 24 and 36. The results demonstrated a significant increase of PPD and BOP values over time for both sites. Particularly, PPD increased for the banded sites after placement until 36 weeks; in the bonded sites, a significant increase in PPD was observed after 18 weeks. Control sites showed no significant changes over time. The authors concluded that the placement of both types of orthodontic attachments has a negative influence on the periodontal variables and microflora; if the inflammation is not controlled, it could have an impact on periodontal health [17].

In 2011, Liu *et al.*, examined changes in periodontal tissues during orthodontic treatment in two groups of young subjects. In the study, periodontal examination (PI, GI, and PPD) was performed before and 1 - 3 months after appliance placement in group A; in group B, periodontal parameters were measured before and 1, 3, and 6 months after appliance removal. The results showed a significant increase in PI and GI, no changes in PPD after the first 3 months of therapy and a decrease in PI, GI, and PPD 6 months after appliance removal. However, at the end of orthodontic therapy, periodontal parameters were higher than those at baseline. The authors concluded that a fixed orthodontic appliance promotes dental plaque accumulation and gingival inflammation, but this observation might be affected by the short-term evaluation period. However, orthodontic appliances seemed to have no permanent effects on periodontal status [23].

In the same year, Van Gastel published a longitudinal study to investigate clinical and microbiological changes after removal of fixed appliances. In this paper, microbiology, PPD and BOP were assessed at baseline (T1), appliance removal (T2) and 3 months post-treatment (T3). Clinical parameters showed a significant increase between T1 and T2 and a decrease between T2 and T3, even if PPD remained significantly higher than T1. The authors concluded that fixed appliances have an impact on microbial and clinical parameters; the periodontal values tended to normalize after de-bonding, but most values remained elevated after de-bonding compared with baseline [21].

In 2014, Ghijselings *et al.* presented a continuation of the study of Van Gastel [21]; this paper added results that were obtained 2 years from fixed appliance removal. The results showed that periodontal parameters increased from baseline to bracket removal and decreased 2 years after treatment. The authors have demonstrated a normalization of clinical parameters, but some periodontal indexes were only partially reversed [22].

The present systematic analysis suggests that there is moderate scientific evidence that fixed appliances influence periodontal status; no articles reported a high score. In conclusion, all articles showed that orthodontic appliances developed generalized plaque accumulation and gingivitis in a short follow-up. Three studies described the long-term potential effects of fixed appliances after de-bonding. Liu *et al.* reported no permanent effects on gingival status [23]. Van Gastel reported that periodontal values tended to normalize 3 months after fixed appliance removal even if the same parameters remained higher with respect to the baseline [21]. Ghijselings concluded that fixed appliance placement does not have a long-term impact on clinical periodontal parameters; in fact, many values normalized 2 years after de-bonding [22].

## CONCLUSION

Orthodontic therapy performed with proper maintenance of oral hygiene could prevent permanent periodontal damage.

Nevertheless, studies conducted on a wider sample size that includes a control group and a longer follow up are needed to obtain statistically significant results regarding the influence of fixed appliances on periodontal health over the long term.

## Figures and Tables

**Table 1:** Methodological quality score.

1. Study design: description of the study design	0.4
2. Participants Sample standards: participant’s inclusion and exclusion criteria. Sample characterization: number and characteristic of participants. Calculation of sample size Control group Ethics: evidence of ethical factors	1.0 0.2 0.2 0.2 0.2 0.2
3. Length of follow-up period 3 a 5 months after bonding ≥ 6 months after bonding After debonding Month collections	4.0 0.5 1.5 1.5 0.5
4. Periodontal outcome measure Periodontal Index (PI) Gingival Index (GI) Bleeding on Probing (BOP) Pocket Probing Depth (PPD)	4.0 0.5 0.5 1.0 2.0
5. Statistical analysis: adequate (indication of the test applied and significance level)	0.2
6. Results: adequate presentation of results (presentation of all proposed results; comparison between results; participant dropout with justification)	0.2

**Table 2:** Quality assessment

**Author/YEAR**	**Study Design**	**Partecipants**						**Length Of Follow Up Period**				**Periodontal Measurement**				**Statistical Analisysis**	**Results**	**Discussion**	**Total Point/Quality**
		Sample standards participant’s	Sample characterization	Calculation of sample size	Control group		Ethics: evidence of ethical factors	2 a 4 months after bonding	≥ 4 months after bond	After debonding	Month collections	PI	GI	BOP	PPD				
Paolantonio *et al* 1999	0	0.2	0.2	0	0	0		0.5	0	0	0.5	0	0	1.0	2.0	0.2	0.2	0.2	5 / low
Ristic *et al.* 2007	0.4	0.2	0.2	0	0	0.2		0	1.5	0	0	0.5	0.5	1.0	2.0	0.2	0.2	0.2	7,1 / moderate
Ghijselings *et al.* 2014	0.4	0.2	0.2	0	0	0.2		0	1.5	1.5	0.5	0	0	1.0	2.0	0.2	0.2	0.2	8.1 / moderate
Van Gastel *et al.* 2008	0.4	0.2	0.2	0	0	0.2		0	1.5	0	0.5	0	0	1.0	2.0	0.2	0.2	0.2	6,6 / moderate
Kaygisiz *et al.* 2015	0	0.2	0.2	0	0.2	0.2		0.5	0	0	0	0.5	0.5	1.0	2.0	0.2	0.2	0.2	5,9 / low
Liu H *et al.* 2011	0	0.2	0.2	0	0	0		0.5	0	1.5	0	0.5	0.5	0	2.0	0.2	0.2	0.2	6 / moderate
Naranjo *et al*. 2006	0	0.2	0.2	0	0.2	0.2		0.5	0	0	0	0.5	0.5	1.0	2.0	0.2	0.2	0.2	5,9 / low
Van Gastel *et al.* 2011	0.4	0.2	0.2	0	0	0.2		0	1.5	1.5	0	0	0	1.0	2.0	0.2	0.2	0.2	7.6 / moderate

**Table 3:** Detailed quality information of studies included in the review.

**Authors/** **Year**	**Study Design**	**Sample Description**	**Total Dtudy Time/Interval Times**	**Periodontal Index**	**Statistical Analysis**	**Conclusion**
**Ristic M / 2007**	Prospective longitudinal controlled study	32 subjects, 13 males and 19 female (from 12 to 18 years old)	before placement of fixed appliance 1, 3, 6 months after	PI, GI, PPD, GBI	Student’s *t-*test and chi-squared test combined with McNemar test	In adolescents, treatment increased the value of periodontal indices.
**Liu H / 2011**	Not mentioned	Group A (at the beginning of treatment) 28 subjects, 22 females, 6 males (17.6+- 5.68 y) Group B (at the completation of treatment) 20 subjects 13 female, 7 males (17.8± 4.49 y)	Group A Before placement of appliance and 1, 3 months after Group B Before appliance removal and 1, 3,6 month after appliance removal	PI, GI, PPD	SPPS 16.0 for statistical analysis, T-test and Spearman test	Fixed orthodontic treatment is conducive to dental plaque accumulation and gingival infiammation. After removal of orthodontic appliances the periodontal condition improved.
**Van Gastel J / 2008**	Longitudinal split-mouth desisgn	24 subjects, 10 boys and 14 girls (14.6± 1.1 years)	Before placement appliance and 20, 24, and 36 weeks after	PPD, BOP	A linear mixed model was used with the data, using time, type, and their interaction as fixed factors.. Multiple comparisons between types and times were set up and a comparison of times was also performed for two types of subgroups.	Placement of both types of orthodontic attachments had a negative influence on the microflora and the clinical periodontal variables
**Ghijselings E / 2014**	Longitudinal prospective design	24 subjects, 10 males, 14 females (14.6±1.1 y)	Before placement of appliance, after bracket removal and 2 years post-treatment	PPD and BOP	A linear mixed model was used with the data, using time, type, and their interaction as fixed factors.. Multiple comparisons between types and times were set up and a comparison of times was also performed for two types of subgroups	Normalization toward the values at baseline was seen 2 years after removal of appliances
**Van Gastel J/** **2011**	Longitudinal prospective design	24 subjects, 10 males, 14 females (14.6±1.1 y)	Before placement of appliance (T1), after bracket removal (T2) and 3 months post-treatment (T3).	PPD and POB	A linear mixed model was used with the data, using time, type, and their interaction as fixed factors.. Multiple comparisons between types and times were set up and a comparison of times was also performed for two types of subgroups	Clinical parameters PPD, POB, and GCF flow showed a significant increase between T1 and T2. Between T2 and T3 these variables decreased significantly but remained significantly higher than at T1 (except for BOP values at the bonded sites)

**Table 4:** Periodontal Index of the 5 selected articles. W (week); CV (coefficient of variation); CI (Confidential Interval); *P < 0.05, **P < 0.01, ***P < 0.001.

–	**Plaque Index** **(PI)**	**Gingival Index(GI)**	**Bleeding on Probing** **(BOP)**				**Pocket Probing Depth** **(PPD)**			
**Ristic 2007** **Tx** first appointment **T0** 3 W later before treatment **T1** 1 month after bracket placement **T3** 3 months after bracket placement **T6** 6 months after bracket placement	(mean± SD;CV %) 1.281± 0.310; 24.110 0.898± 0.329; 36.637 1.211± 0.278; 22.956 1.250± 0.336; 26.880 1.219± 0.275; 22.560	(mean± SD;CV %) 0.586± 0.288; 49.147 0.383± 0.269;70.235 1.148± 0.310; 27.003 1.352± 0.430; 31.805 1.305± 0.380; 29.119	(mean± SD;CV %) 0.516± 0.416; 80.620 0.266 ± 0.269 ;101.128 1.320 0.586; 44.394 1.336± 0.677; 50.674 1.383± 0.453 ; 32.755				(mean± SD;CV %) 2.500± 0.412; 16.480 2.500± 0.386; 15.440 3.039± 0.436; 14.347 3.211± 0.550; 17.129 3.188± 0.557; 17.472			
**Van Gastel 2008** **T0** (molar band placement) **T1** W 18 (brackets placement) **T2** W 20 **T3** W 24 **T4** W 36	–	–	(mean ± SD) **Banded sites** 0.34 ±0.05 0.47 ±0.06 1.57 ±0.04* *P<0.05 value banded vs control sites (Tx/T 0)	(mean ± SD) **Bonded sites** 0.24 ±0.04 0.21 ±0.01 0.53 ±0.03* 0.59 ±0.02* 0.89 ±0.03* *P value for bonded sites (T x/ T1)		(mean ± SD) **Control sites** 0.12± 0.02 0.22± 0.09 0.16± 0.01	(mean ± SD) **Banded sites** 1.85 ±0.05 2.49 ±0.03* 2.98 ±0.04* *P<0.05 value banded vs control sites (Tx/T 0)	(mean ± SD) **Bonded sites** 2.00 ±0.04 2.14 ±0.01 2.49 ±0.02* 2.62 ±0.02* 2.94 ±0.02* *P value for bonded sites (T x/ T1)		(mean ± SD) **Control sites** 2.04 ±0.02 2.23 ±0.01 2.28 ±0.02
**Liu H / 2011** **T0** before therapy **T1** 1 month after treatment start **T2** 3 months after treatment start **T4** before appliance removal **T5** 1 month after removal **T6** 3 months after removal **T7** 6 months after removal	(mean ± SD) 0.36 ± 0.45 0.66 ±0.41* 0.87 ± 0.46* 0.99±0.20 0.63± 0.26* 0.62± 0.29* 0.64± 0.24*	(mean ± SD) 0.29 ± 0.54 0.96 ± 0.51* 0.96 ± 0.43* 1.7 ± 0.73 1.0 ± 0.56 0.5 ± 0.51* 0.45 ± 0.61*	–				(mean ± SD) 1.05 ± 0.11 1.17 ± 0.19 1.11 ± 0.22 1.68 ± 0.22 1.544 ± 0.24 1.49 ± 0.22* 1.41 ± 0.23*			
**Van Gastel J / 2011** **T1** before the attachments placement **T2** at bracket removal **T3** 3 months after bracket removal **T1** is baseline (T-18 for the headgear group, T0 for the non-headgear group)	–	–	(mean ± SD) **Banded sites** 0.4 ± 0.3 * 1.7 ± 0.4 ** 1± 0.5 ***		(mean ± SD) **Bonded sites** 0.2 ± 0.1 * 1.2 ± 0.3 ** 0.5 ± 0.2 *		(mean ± SD) **Banded sites** 2.1± 0.2 * 3.7± 0.5 ** 2.9± 0.25 ***		(mean ± SD) **Bonded sites** 2.2 ± 0.2 * 3.25 ± 0.1 ** 2.8 ± 0.1 ***	
**Ghijselings E / 2014** **T1** before the attachments placement **T2** at debonding **T3** 2 years after debonding **T1** is baseline (T-18 for the headgear group, T0 for the non-headgear group)	–	–	(mean;CI) **Banded sites** 0.357; 0–0.714 1.738; 1.381– 2.095 0.794; 0.424–1.165 T1-T2 (p<0.05) for banded sites T3-T1 (p < 0.05) for banded sites		(mean;CI) **Bonded sites** 0.248; 0–0.504 1; 1– 1.3 0.396; 0.128-0.664 T1-T2 (p<0.05) For bonded sites		(mean ± SD) **Banded sites** 2.1± 0.4 3.7± 0.5 2.1± 0.2 T1-T2 (p < 0.05) for banded sites		(mean ± SD) **Bonded sites** 2.2± 0.1 3.25± 0.2 2.0± 0.1 T1-T2 (p < 0.05) for bonded sites	
